# Miliary Tuberculosis Presenting as Puerperial Fever

**DOI:** 10.1155/2011/893515

**Published:** 2011-06-30

**Authors:** A. Agarwal

**Affiliations:** Military Hospital (CTC), Golibar Maidan Pune 411040, India

## Abstract

Miliary tuberculosis as a cause of puerperial fever is extremely rare. It is a serious illness with nonspecific clinical manifestations and typical chest radiographic findings may not be seen until late in the course of the disease. It is often associated with maternal immunocompromised status. Here, we report a case of miliary tuberculosis in a nonimmunocompromised mother presenting as fever of unknown origin in immediate puerperium. Prolonged workup of eight weeks led to the diagnosis of miliary tuberculosis as the cause of postpartum fever that responded well to antituberculous drugs.

## 1. Introduction

Miliary tuberculosis is a potentially lethal form of tuberculosis resulting from massive lymphohematogenous dissemination of *Mycobacterium tuberculosis* bacilli. Its incidence is estimated at approximately 1% of all tuberculosis cases [1]. It is uncommon during pregnancy, and diagnosis tends to be delayed as symptoms are apt to be mixed up with those for pregnancy and puerperium. The disease is often associated with a maternal history of intravenous drug abuse, malignancy, alcoholism, or human immunodeficiency virus infection. This article reports a rare case of miliary tuberculosis presenting as fever of unknown origin in immediate puerperium without any of these risk factors.

## 2. Case History

A 28-year-old female gravid- 2 Para- 1 with an uneventful antenatal period delivered a full-term female child weighing 2.5 Kg by normal vaginal delivery. She developed intermittent high-grade fever associated with chills one week after delivery. There were no other complaints. There was no significant past history of illness or contact with a case of tuberculosis. General physical examination revealed pallor. There were no enlarged lymph nodes or palpable liver and spleen. The lungs were clinically normal. Initial laboratory investigations were as follows: haemoglobin-8.3 gm/dL, MCV-96.5 fl, MCH-28.2 pg, and MCHC-29.2 g/dL, total leucocyte count was 7,500/cumm with 57% polymorphs and 30% lymphocytes. Erythrocyte sedimentation rate (Westergreen) was 45 mm fall first hour. Liver and renal function tests were within normal limits. Widal test for enteric fever, strip test for malarial parasite, and urine and blood culture were not contributory. Serology for HIV, HBV, and HCV was negative. Chest radiography and ultrasound abdomen done during second week of fever were essentially normal.

Patient was initially treated as a case of viral fever and then empirically for enteric fever. She, however, continued to have low-grade fever with night sweats. Three weeks later follow-up ultrasound abdomen revealed mild hepatosplenomegaly. Repeat chest radiograph done at this stage was normal. Mantoux test and mycobacteriology serology panel for Anti-A60 Mycobacterium IgG, IgA, and IgM were negative. Patient was now put empirically on antimalarials but she continued to be symptomatic.

**Figure 1:**
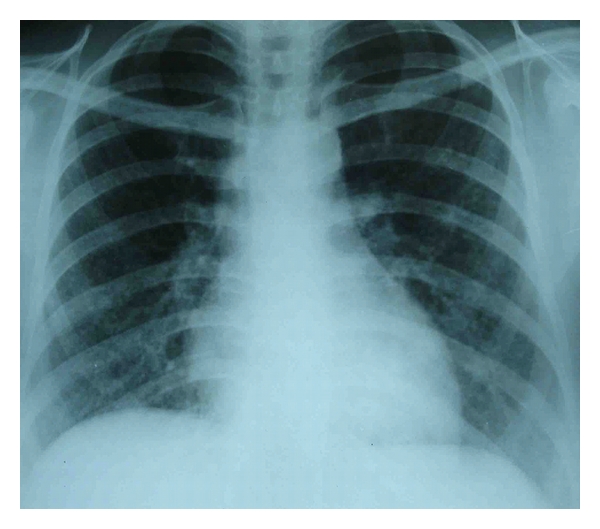
Chest radiograph showing bilateral diffuse miliary nodular lesions.

**Figure 2:**
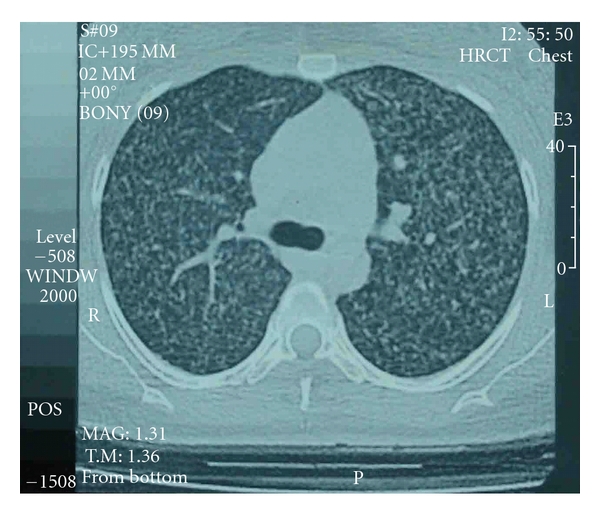
HRCT chest-lung window: bilateral diffuse miliary nodular opacities showing random distribution.

Chest radiograph repeated four weeks later suggested the presence of bilateral miliary mottling (Figure 1). The diagnosis of miliary tuberculosis was confirmed by high resolution CT scan (Figure 2). The patient was started on 4-drug regimen ATT (isoniazid, rifampicin, ethambutol, and pyrazinamide) for first three months, followed by isoniazid and rifampicin for next six months. In a few days there was significant improvement in general condition of the patient. She became afebrile after 10 days of ATT. Followup imaging study after 9 month of treatment revealed no residual activity. Newborn was vaccinated with BCG at birth. She was breast fed and put on INH prophylaxis for 3 months. She had a normal chest skiagram and negative Mantoux test before and after INH therapy. Both mother and child are doing fine after one year of follow-up.

## 3. Discussion

Views as to whether the incidence of tuberculosis is increased by pregnancy have varied over time. The Hippocratic view that pregnancy was beneficial to tuberculosis was generally held until the 19th century [2]. By the early 20th century opinion had swung to pregnancy having a deleterious effect on tuberculosis, so much so that abortion was recommended [3]. Data from San Domingo gave no evidence that pregnancy increased the chance of tuberculosis developing postpartum in either HIV- positive or-negative women [4]. Now, tuberculosis is believed to get flared up by the stress of pregnancy, especially in association with a poor nutritional status, immunodeficient state, or co-existent diseases. The loss of protective antibodies in mother during lactation too favours the development of postpartal tuberculosis. However, more studies are needed to substantiate the hypothesis [5].

The term “miliary tuberculosis” refers to all types of progressive disseminated hematogenous tuberculosis regardless of the pathological picture [6]. It is a curable disease, yet fatal if left untreated, and, therefore, prompt diagnosis is mandatory. The disease often presents as a fever of unknown origin (FUO) [6]. Other symptoms include malaise, weakness, anorexia, weight loss, and night sweats. In one, third of patients, hepatomegaly or splenomegaly can be found [6] as was in our case. Hematological abnormalities such as chronic disease anemia, leucopenia with lymphopenia, thrombocytopenia or, more rarely, pancytopenia, are frequently described [6]. 

Miliary tuberculosis is usually diagnosed when miliary infiltrates are found either on chest roentgenograms and CT scans or when there are autopsy or biopsy findings of miliary organ involvement [6]. However, chest radiography may or may not reveal miliary infiltrates, as such lesions need weeks to develop or may not develop at all [6]. High-resolution computed tomography (HRCT) is more sensitive than plain chest radiography in detecting this disease both early and accurately in its course [7]. 

The determinants of successful dissemination include state of host cellular immunity. Our patient neither had HIV infection nor an apparent cause of immunosuppression. Negative Mantoux test may suggest impaired cellular immunity. However, false negative mantoux test and tuberculosis serology may be seen early in the course of tuberculosis or with severe systemic disease such as meningeal or miliary tuberculosis. 

The same regimens are recommended for use in pregnancy and lactation as for the nonpregnant state. Use of Isoniazid, Rifampicin, Ethambutol, Pyrazinamide, Streptomycin, Kanamycin, and Cycloserine has been considered safe for breast feeding, but safety of PAS is unproven. Under RNTCP, breast feeding of neonates is recommended regardless of the mother's TB status [5].

Neonates born to mothers having infectious tuberculosis should be given chemoprophylaxis with INH for 3 months or till the mother becomes noninfectious. BCG vaccination may be postponed or done with INH-resistant BCG vaccine. After 3 months, if the mother has a negative sputum smear and the neonate (with a normal chest skiagram) has a negative Mantoux test, then INH chemoprophylaxis may be discontinued. In case the Mantoux test is positive, a thorough search should be made for locating the presence of pulmonary or extrapulmonary focus and administration of ATT may be decided accordingly [5].

This case is reported to emphasize that high index of suspicion is required for early diagnosis and timely management of this curable, yet potentially fatal disease. Clinicians usually include tuberculosis in their differential diagnosis list when managing patients with fever of unknown origin. However, due to non-specific clinical manifestations and absence of typical chest radiographic findings, miliary tuberculosis as an etiology is usually bypassed and the diagnosis (if any) is delayed.
